# Microstructure and Shape Memory Properties of Gas Tungsten Arc Welded Fe-17Mn-5Si-10Cr-4Ni-(V, C) Shape Memory Alloy

**DOI:** 10.3390/ma17184547

**Published:** 2024-09-16

**Authors:** Dohyung Kim, Taeyoon Kim, Changwook Ji, Sangwon Ji, Wookjin Lee, Wangryeol Kim

**Affiliations:** 1 School of Materials Science and Engineering, Yeungnam University, Gyeongsan 38541, Republic of Korea; dhyungkim@pusan.ac.kr; 2 School of Materials Science and Engineering, Pusan National University, Busan 46241, Republic of Korea; nanitz@kitech.re.kr; 3 Dongnam Regional Division, Korea Institute of Industrial Technology, Yangsan 50623, Republic of Korea; 4 Ulsan Regional Division, Korea Institute of Industrial Technology, Ulsan 44776, Republic of Korea; cwji@kitech.re.kr; 5 Department of Civil Engineering, Chungbuk National University, Chungju 28644, Republic of Korea; tkddnjs0727@chungbuk.ac.kr

**Keywords:** Fe-based shape memory alloy, gas tungsten arc welding, digital image correlation, phase transformation

## Abstract

In this study, microstructure, mechanical, and shape memory properties of the welded Fe-based shape memory alloy (Fe-SMA) plates with a nominal composition of Fe-17Mn-5Si-10Cr-4Ni-(V, C) (wt.%) by gas tungsten arc welding were investigated. The optimal heat input to ensure full penetration of the Fe-SMA plate with a thickness of 2 mm was found to be 0.12 kJ. The solidified grain morphology adjacent to the partially melted zone was columnar, whereas the equiaxed morphology emerged as solidification proceeded. The ultimate tensile decreased after welding owing to the much larger grain size of the fusion zone (FZ) and heat-affected zone (HAZ) than that of the base material (BM). Weldment showed lower pseudoelastic (PE) recovery strain and higher shape memory effect (SME) than those of the plate, which could be ascribed to the large grain size of the FZ and HAZ. Recovery stress (RS) slightly decreased after welding owing to lower mechanical properties of weldment. On the other hand, aging treatment significantly improved all PE recovery, SME, and RS via carbide precipitation. Digital image correlation analysis revealed that HAZ showed the lowest SME after heating and cooling, implying that the improved SME of FZ compensated for the low SME of the HAZ.

## 1. Introduction

Fe-based shape memory alloy (Fe-SMA) is a promising metallic material exhibiting unique functional properties, such as a shape memory effect (SME), pseudoelasticity (PE), and recovery stress (RS). SME and PE are types of shape recovery from a deformed state. The former is activated by heating above the austenite transformation starting temperature (As), and the latter proceeds immediately after removing the load. Since Sato et al. observed large SME in an Fe–30Mn–1Si single crystal [1], numerous attempts have been made to improve the shape memory properties of polycrystalline Fe-SMAs [2,3,4,5].

Fe-SMAs have attracted attention in numerous civil engineering applications owing to their affordability compared with Ni–Ti- and Cu-based alloys. Attributed to the unique functionalities of Fe-SMAs, numerous studies have attempted to explore their applications in seismic dampers, prestressing units for concrete, and pipe coupling [6,7,8,9,10]. Although the crucial PE property of Fe-SMAs is required for seismic damping units to absorb energy, prestressing and pipe-coupling applications require substantial RS. Leinenbach et al. developed Fe–17Mn–5Si–10Cr–4Ni–(V, C) (wt.%) alloy exhibiting substantial RS [11]. Subsequently, Yang et al. developed a two-step aging process for maximizing RS by controlling the size and distribution of vanadium carbide (VC) precipitates [12].

Pipe coupling is one of the promising applications that require substantial RS and SME. Fe-SMAs are typically transformed into tube-shaped components to be used as pipe couplers. Although several methods for fabricating the tube have been reported, piercing and cutting to fabricate a seamless tube are generally considered expensive and ineffective [13]. Rather, welding after bending has been proposed as an effective alternative. Druker et al. investigated the microstructure and shape memory properties of welded Fe–15Mn–5Si–9Cr–5Ni by gas tungsten arc welding (GTAW) [14]. They also reported the effect of heat treatment on the shape memory properties of weldment and a proper bending methodology. Liu et al. reported the microstructure of laser-welded Fe–17Mn–5Si–10Cr–5Ni, although they did not demonstrate the shape memory performance [15]. Dong et al. compared the shape memory properties of Fe–28Mn–6Si–5Cr–0.53Nb–0.06C alloys fabricated by laser-beam welding (LBW), electron-beam welding, and tungsten inert gas welding [16]. They reported that LBW displayed the best shape memory performance, although its performance slightly deteriorated regardless of the employed welding process. The extant studies noted similar post-welding deteriorations of shape recovery performances, and even though the microstructures and shape memory properties of various SMA weldments have been determined, the causes of such deteriorations have not been clarified. Moreover, welding of age-hardenable VC containing Fe-Mn-Si SMA has not been studied yet in the literature. Furthermore, the local differences in shape memory properties of fusion zone (FZ) and heat-affected zone (HAZ) also should be investigated because the microstructure and corresponding properties of FZ and HAZ is different [17]. 

In this study, the microstructure and shape memory properties of Fe–17Mn–5Si–10Cr–4Ni–(V, C) (wt.%) alloy weldments fabricated by GTAW were investigated to give an basic understanding of welding of VC carbide containing Fe-Mn-Si SMA. The SME and RS of the weldment fabricated under optimized welding conditions were measured. In addition, to reveal the reason why the shape memory properties are varied when Fe-SMAs are welded, the local strain was measured during heating (the activation of shape recovery) by digital image correlation (DIC). Subsequently, microstructural features of the fusion zone (FZ) and heat-affected zone (HAZ) were separately characterized in a fully recovered state. Furthermore, the effect of post-aging on shape memory properties was also investigated.

## 2. Materials and Methods

The base material (BM) used in this study was an Fe-SMA plate with 2 mm thickness and a nominal composition of Fe–17Mn–5Si–10Cr–4Ni–(V, C) (wt.%) provided by refer AG, Switzerland. GTAW was employed to weld the plate in the pulse mode without a filler material. The flow rates of the Ar gas flow for shielding and purging were fixed at 20 and 10 L/min, respectively, and the voltage, arc torch angle, and pulse frequency were fixed at 10 V, 10°, and 5 Hz, respectively. To optimize the welding condition, the arc current and welding speed were varied in the heat input range of 0.08–0.12 kJ/mm (Table 1). The two values of current in the form of A/B mean maximum and minimum current of the pulsed current. The heat input was calculated as follows:

(1) Q=P/v=(EI)/v,

where, Q, P, E, I, and v are the heat input, electrical power, voltage, arc current, and welding speed, respectively.

The microstructures of the weldments were characterized by optical microscopy (OM, Eclipse E200, Nikon, Minato City, Japan) and scanning electron microscopy (SEM, JSM7200F, JEOL, Tokyo, Japan), equipped with energy-dispersive spectroscopy (EDS, X-MaxN, Oxford Instrument, Abingdon, UK). Electron backscatter diffraction (EBSD, Nordlysnano, Oxford Instrument, Abingdon, UK) analysis was performed to further investigate the phase evolution, grain boundary (GB) characteristics, and local misorientation of the weldments. The low-angle GBs (LAGBs) and high-angle GBs (HAGBs) were defined as cases where the neighboring grain-misorientation angles were 2–15° and >15°, respectively. Further, kernel average misorientation (KAM) maps were obtained in the local misorientation range of 0–5°. The software, CHANNEL 5 5.0, was used to process the EBSD data. The samples for the microstructure analysis were ground using SiC paper, followed by polishing using alumina and colloidal silica suspensions. The samples for the OM analysis were etched with a mixed solution of hydrochloric acid, nitric acid, and distilled water in a 2:1:1 proportion.

Mechanical and shape memory properties were measured by a universal testing machine (UTM, Instron 8516, Instron, Norwood, MA, USA). The flat-type dog-bone-shaped specimens were machined according to the ASTM E8 subsize. Further, the stress–strain curve for the tensile properties was obtained at a displacement rate of 0.5 mm/min. The mechanical behavior was monitored by a strain gauge at the center of the specimen. To characterize the SME, the recovery strain was measured as follows: first, a tensile prestrain of 4% was applied to the sample, followed by unloading to <5 MPa. Thereafter, the sample was heated to 200 °C by an electrical heating system and cooled to room temperature (25 °C) while maintaining the applied stress at <5 MPa. The RS, i.e., the stress generated by SME upon heating under restrained conditions, was measured by the same procedure as in the recovery strain measurement, although unloading was conducted to 5 MPa, and the strain, rather than the applied stress, was kept constant during the heating and cooling cycles. To evaluate PE, the samples were loaded up to 4% of the prestrain and unloaded to a holding stress of <5 MPa. The PE was defined by the difference between the hypothetic strain upon linear unloading and the experimentally measured strain upon unloading, as follows:

(2) $$PE=\left(\left(\varepsilon_{max}-\varepsilon_{min}\right)-\frac{\sigma_{max}}{E}\right)\times 100$$

where ε_max_ and ε_min_ are the strains before and after unloading, respectively; σ_max_ is the stress measured at 4% strain; E is the elastic modulus. Here, E was evaluated by extrapolation using the stress–strain dataset in the range of 0–0.05%. The total recovery is calculated of summation of PE and SME. The ε_max_ − ε_min_ means recovery strain after unloading without heating and σ_max_/E means elastic recovery strain. Therefore, the subtraction of the two values gives us recovery strain originated from phase transformation, PE. The DIC analysis was performed on Ncorr 2D-DIC software v1.2.2 implemented in MATLAB to reveal the local recovery strain of the weldment [18]. The Vickers microhardness (Hv) was measured as the average of five separate tests at the surface of each specimen under a load of 0.1 kgf (HM-220A, Mitutoyo Corp., Kawasaki, Japan).

The two-step aging treatment proposed by Yang et al. was conducted to investigate the effect of post-heat treatment on the mechanical and shape memory properties of the weldments [12]. They established a two-step heat treatment condition to maximize RS by controlling the size and distribution of the VC precipitates. The first step comprised a heat treatment at 600 °C for 20 h to ensure the maximum number density of the nuclei, and the second step proceeded at 670 °C for 6 h to facilitate the growth of the precipitates.

## 3. Results and Discussion

**Figure 1:**
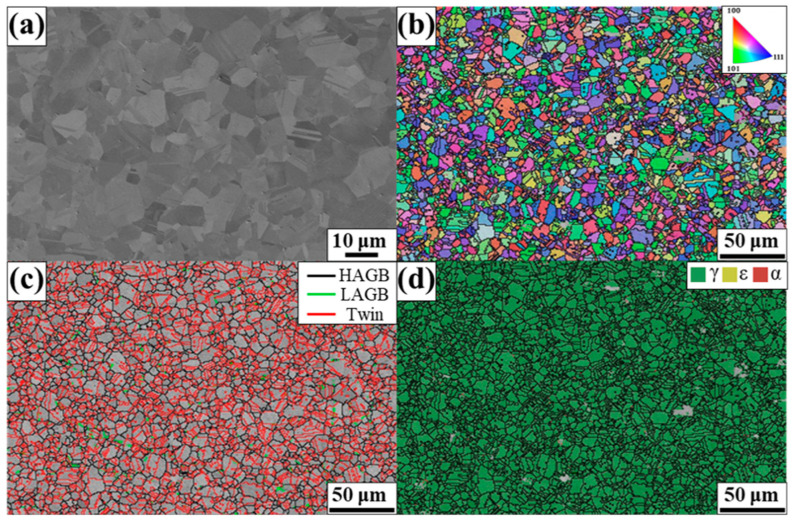
Microstructure of the BM. (**a**) Secondary electron (SE) image, (**b**) inverse pole figure (IPF) map, (**c**) GB map, and (**d**) phase map.

Figure 1 shows the microstructure of BM composed of Fe–17Mn–5Si–10Cr–4Ni–(V, C) (wt.%). The SE image and IPF map indicate the fully recrystallized grain structure without a specific crystallographic texture (Figure 1a,b). The GB map in Figure 1c presents a high fraction of the twin boundary with an insignificant amount of LAGB. Figure 1d shows the phase map, displaying the fully austenitic structure with a face-centered cubic structure.

**Figure 2:**
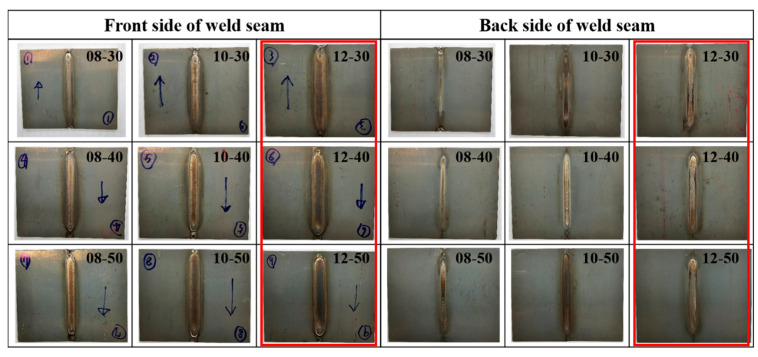
Pictures of the front and back sides of the weld seams formed under various welding conditions.The arrows indicate welding direction. The three samples decorated by red square had fully penetrated weld seam.

Figure 2 shows the weld seam condition captured from the front and back sides of the weldments formed under various welding conditions. Regardless of the welding speed, all conditions below a heat input of 0.12 kJ/mm do not ensure full penetration, i.e., do not ensure the formation of the back-side weld seam, whereas the conditions with a heat input of 0.12 kJ/mm, i.e., 12-30, 12-40, and 12-50, exhibited fully penetrated weld seam.

**Figure 3:**
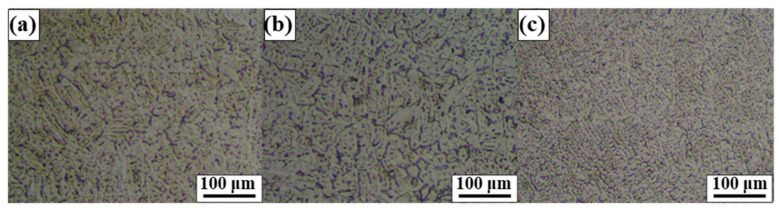
OM images of the center of the FZ of (**a**) 12-30, (**b**) 12-40, and (**c**) 12-50.

The microstructures of the weld seams formed by three different weld conditions were further investigated to select the optimal condition. Figure 3 shows the OM images of the three samples exhibiting fully penetrated weld seams. The samples exhibited typical dendritic microstructures similar to those reported by the extant studies on Fe-SMA welding by GTAW [16]. However, samples 12-50 showed much finer dendritic structure. The secondary dendrite arm spacing (SDAS) can be estimated as follows [19]:

(3) $$\lambda_{2}=k_{1}t_{1s}^{n}=k_{1}{\left(\Delta T/\emptyset \right)}^{n}$$

where λ_2_, k_1_, t_1s_, n, ΔT, and ∅ represent the SDAS, constant depending on the material, solidification time, empirical constant, solidification temperature range, and cooling rate, respectively. As expressed in Equation (3), SDAS became finer as the cooling rate increased. In this case, assuming k_1_ and ΔT are identical for all three cases, SDAS might depend on the cooling rate because the same material was employed for welding. The higher welding speed leads to higher cooling rate because the heat to form a molten pool moves away more quickly. In other words, when the heat source moves away quickly, the region being solidified is less affected by progressing heat source. Although the highest current was applied to compensate for the increased welding speed, i.e., to identify the heat input for all samples, the highest welding speed could induce the finest SDAS, resulting in a fine dendritic structure.

**Figure 4:**
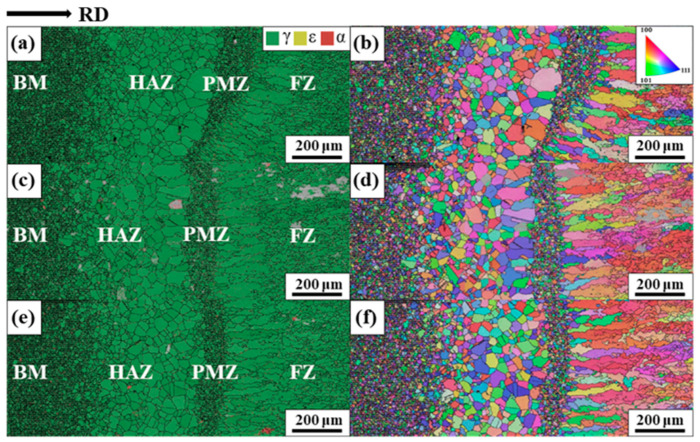
Phase and IPF maps of the samples with full penetration. (**a**,**b**) 12-30, (**c**,**d**) 12-40, and (**e**,**f**) 12-50.

Figure 4 shows the phase and IPF maps of the samples with full penetration. Figure 4 shows the well-separated FZ, HAZ, partially melted zone (PMZ), and BM. Figure 4a,c,e show the fully austenitic structure with negligible amounts of ferrite in the FZ, HAZ, and PMZ. Ferretto et al. reported that the primary solidification phase of an alloy with the same composition as that in this study is δ-ferrite, with a body-centered cubic structure [20]. However, the sample exhibiting the same chemical composition as that used in this study but was fabricated by a laser powder bed fusion (L-PBF) process exhibited a fully austenitic structure. The fully austenitic structure was attributed to the intrinsic heat treatment effect during consecutive laser process during building. They explained that the cyclic heat exposure caused phase transformation from primarily solidified δ-ferrite to austenite. Conversely, Kim et al. presented a dual-phase structure comprising δ-ferrite and austenite when the same material was fabricated by L-PBF but under different building conditions [21]. Both phases exhibited cellular solidification structure, indicating that both were primary phases. Therefore, the crystal structure after solidification highly depended on the environment during solidification.

**Figure 5:**
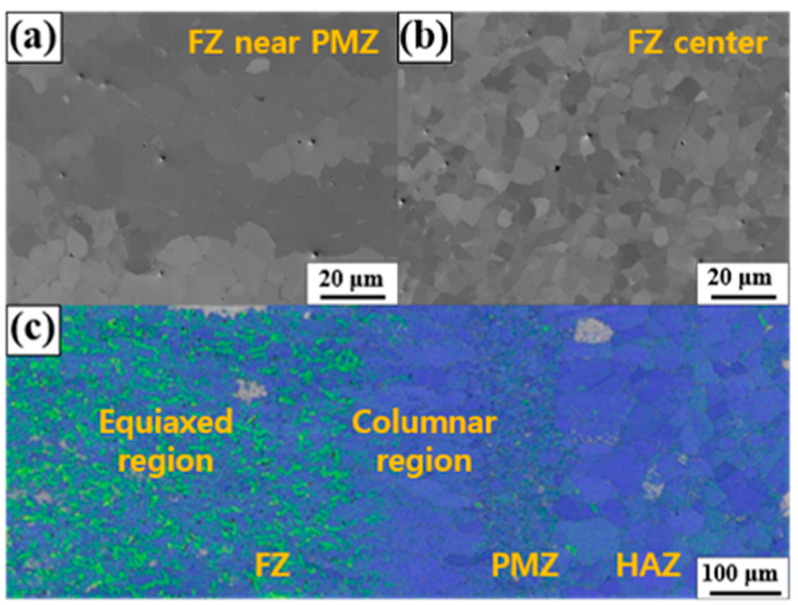
(**a**,**b**) BSE images and (**c**) KAM map of the 12-50 sample. (**a**) FZ near PMZ, (**b**) FZ center.

Generally, GTAW exhibits much lower cooling and solidification rates than L-PBF, as melt pools formed by GTAW are much larger than those formed by L-PBF, although the linear or volumetric heat input of L-PBF is not much higher than that of GTAW. Nagira et al. observed the solidification behavior of Fe–Mn–Si-based SMA during tungsten inert gas spot welding using synchrotron X-ray [22]. They reported that columnar dendritic δ-ferrite was primarily solidified in the early stage of solidification of Fe–15Mn–11Cr–7.5Ni–4Si (wt.%) alloy. Subsequently, equiaxed dendritic austenite was formed by the partitioning of Ni into the liquid phase and the decreased ratio between the thermal gradient and solidification velocity; finally, the δ-ferrite formed in the early stage was transformed into austenite in the final stage. In this study, the solidification seemed to proceed by a path proposed by Nagira et al. [22]. Figure 5 shows the backscattered electron (BSE) images and KAM map of the 12-50 sample. Figure 5a,b shows the grain morphologies of the center of FZ and the region adjacent to PMZ. Figure 5a shows the columnar grain morphology, whereas Figure 5b shows the equiaxed morphology. The difference in grain morphology further supports the solid state phase transformation during solidification. Figure 5c shows a negligible local misorientation near PMZ, i.e., the early solidified region, and relatively high local misorientation at the region far away from the molten pool boundary. The negligible local misorientation near the PMZ presumably resulted from the atomic rearrangement during solid–solid phase transformation from primary δ-ferrite to austenite.

Figure 4b,d,f show the IPF maps of the samples with full penetration. The coarsened grains in HAZ did not exhibit a clear crystallographic texture similar to BM. The average widths of the HAZ for 12-30, 12-40, and 12-50 samples were 440.38, 456.04, and 405.49 μm, respectively, indicating that there was no significant difference in the average HAZ widths, although the HAZ of Sample 12-50 was slightly narrow. The grains with the same or similar crystallographic orientations tended to be divided into several subgrains as the solidification proceeded, supporting the change in the solidification mode from columnar dendrite to equiaxed dendrite.

**Figure 6:**
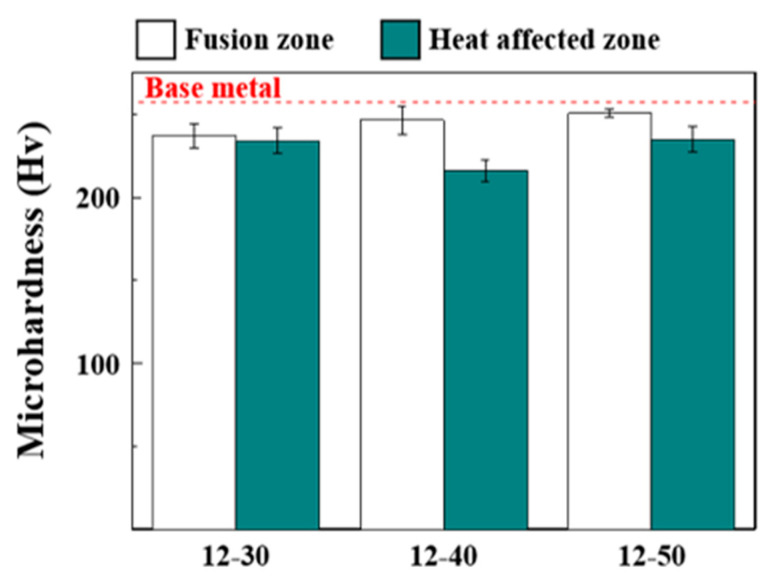
Vickers microhardness of the samples with full penetration.

Figure 6 shows Vickers microhardness of the samples with full penetration. All samples exhibited similar hardness values; the values measured from FZ and HAZ were lower than those from BM. The lower hardness values of HAZ and FZ could be attributed to the enlarged grain structure and larger grain size than BM, respectively. However, the hardness values measured from FZ were generally higher than those from HAZ. The possible reason is that each grain having same crystallographic orientation was subdivided by the equiaxed dendrites with local misorientation, facilitating the refinement strengthening effect, as would be further discussed. The higher number of precipitates in FZ than in HAZ can also be another reason for higher hardness via precipitation hardening. The precipitation behavior will also be discussed later. 

Combining the results from Figure 2, Figure 3, Figure 4, Figure 5 and Figure 6 revealed that there were no noticeable differences in the microstructures, HAZ widths, and microhardness of the three samples with full penetration. The authors selected 12-50 as the optimal-condition sample as a high welding speed is desirable for high productivity. Obviously, further experiments are needed to find the best welding conditions. However, the aim of this study is to reveal the relationship between microstructure and physical properties of weldment. Therefore, further work for optimizing welding conditions will be reported in a separate study. Hereafter, the employed welding condition was identical to that of Sample 12-50 unless otherwise noted.

**Figure 7:**
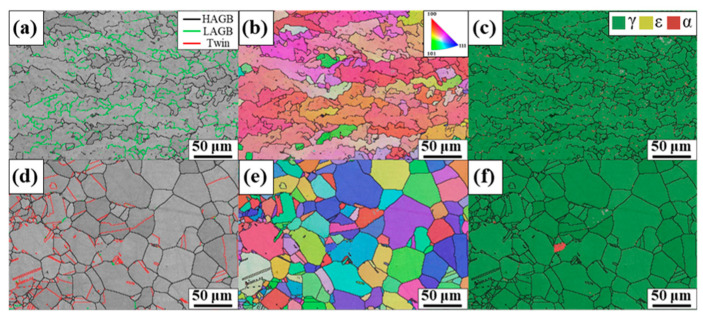
Microstructures of (**a**–**c**) FZ and (**d**–**f**) HAZ. (**a**,**d**) GB, (**b**,**e**) phase, and (**c**,**f**) IPF maps.

Furthermore, FZ and HAZ were investigated. Figure 7a,b show that the grains with the same or similar crystallographic orientations were subdivided by LAGB. Although the region distinguished by HAGB exhibited a columnar morphology, the subgrains divided by LAGB exhibited an equiaxed morphology. Figure 7c shows the fully austenitic structure, with tiny fractions of the ferrite phase presented as red dots. Figure 7d–f show the polygonal grain morphology with a relatively high fraction of twin boundaries and a fully austenitic structure. As investigated above, HAZ and BM exhibited almost the same microstructures, although grain coarsening occurred in HAZ during welding.

**Figure 8:**
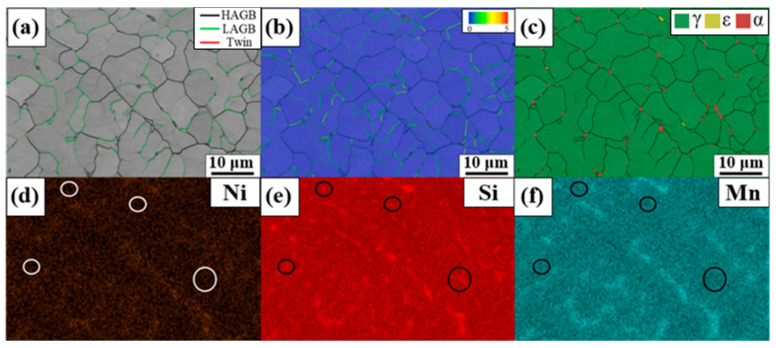
(**a**–**c**) Higher-magnification microstructure of FZ and the (**d**–**f**) EDS mapping. (**a**) GB, (**b**) KAM, (**c**) phase, (**d**) Ni, (**e**) Si, and (**f**) Mn maps. The white and black circles indicate elemental segregation at the triple junctions.

The GB and KAM maps (Figure 8a,b) showed the equiaxed morphology more clearly. The region with relatively high local misorientation (Figure 8b) was almost identical to LAGB in Figure 8a. LAGB and HAGB decorated the equiaxed subgrain boundaries, supporting the austenite equiaxed dendrite-solidification mode in the center of FZ. LAGB dividing the grains decorated by HAGB could exert a grain refinement–strengthening effect because LAGB could act as a soft barrier to movement of perfect dislocations [23]. Therefore, a high LAGB fraction could account for the higher microhardness of FZ than that of HAZ.

A small amount of the ferrite phase was also observed in Figure 8c, and most of them were located on triple junctions of the GBs. Figure 8d–f show the elemental maps of the identical region to Figure 8a–c. As reported by Nagira et al., Mn, Ni, and Si are likely to be segregated in the liquid during solidification [24]. The Mn, Ni, and Si segregations were also confirmed by EDS mapping. Although the regions with elemental segregation were almost identical for the three elements, Si segregation could be further observed at triple junctions indicated by black and white circles in Figure 8d–f. As observed by Gui et al., the partition coefficient of Si increased abruptly in the later solidification stage when the primary solidification phase was austenite [25]. The segregation of Si at the triple junctions was reasonable when the triple junctions were assumed to be formed by the impingement of the growing equiaxed dendrite at the end of solidification. As Si is a ferrite-stabilizing element, the ferrite phase at the triple junctions could be elucidated by Si segregation due to different partition coefficients.

**Figure 9:**
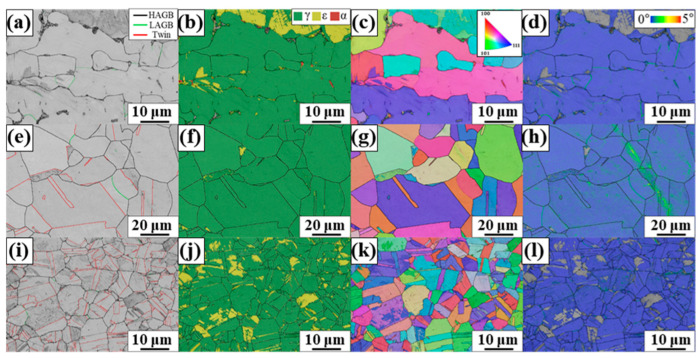
Microstructures of (**a**–**d**) FZ, (**e**–**h**) HAZ, and (**i**–**l**) BM after aging treatment. (**a**,**e**,**i**) GB, (**b**,**f**,**j**) phase, (**c**,**g**,**k**) IPF, and (**d**,**h**,**l**) KAM maps.

Figure 9 shows the microstructures of FZ, HAZ, and BM after aging treatment. Their grain structures did not change significantly owing to low aging treatment temperatures, i.e., 600 °C and 670 °C. However, the ε-martensite phase with a hexagonal close-packed crystal structure was observed in FZ, HAZ, and BM (Figure 9b,f,j), whereas fully austenitic structures were observed before the aging treatment. The extant studies on carbide-containing Fe-SMAs also reported the formation of the ε-martensite phase after heat treatment [20,26]. Kajiwara et al. reported that carbide precipitation induces a lattice misfit between the matrix and carbide, resulting in the formation of stacking faults or ε-martensite [4]. For the alloy system employed in this study, it has been reported that VC and chromium-rich carbide can be formed during aging treatment [20,21].

**Figure 10:**
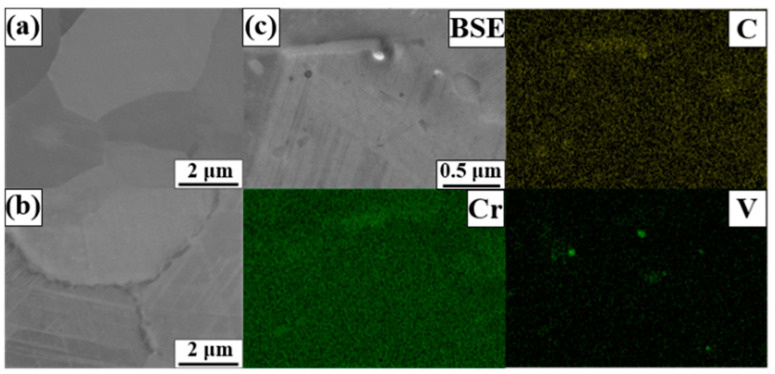
BSE images (**a**) before and (**b**) after the aging treatment. (**c**) EDS mapping after the aging treatment.

Figure 10 also confirms the formation of precipitates after the aging treatment. Figure 10a,b show the microstructures captured from FZ before and after the aging treatment. A comparison of Figure 10a,b indicates that substantial precipitation occurred in GBs after the aging treatment. Figure 10c shows the EDS mapping results, revealing the presence of two kinds of precipitates, one chromium-rich carbide and another VC precipitates, and this correlates with the findings of previous studies [27,28]. Therefore, it can be concluded that the ε-martensite phase was induced by carbide formation.

**Figure 11:**
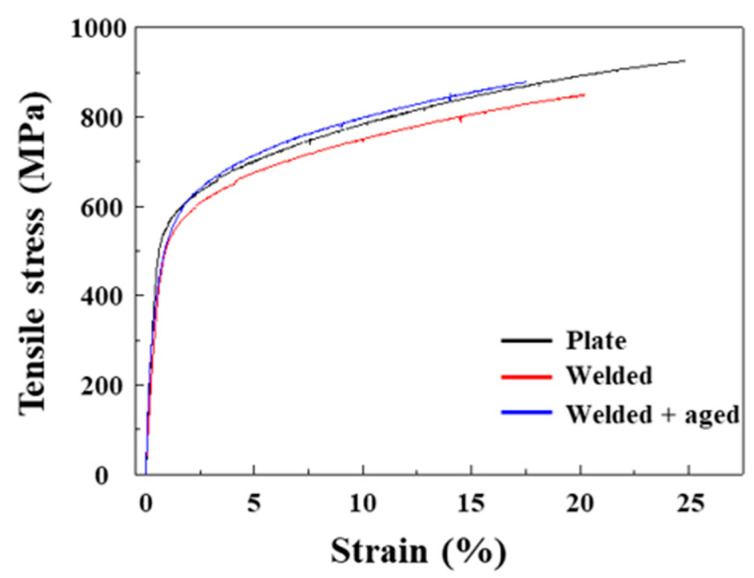
Stress–strain curves obtained from the uniaxial tensile test.

Figure 11 shows the stress–strain curves obtained from a uniaxial tensile test. The nonwelded Fe-SMA plate exhibited the highest ultimate tensile stress (UTS), ~926 MPa. The UTS value decreased to ~850 MPa after welding the Fe-SMA plates. The decrease in UTS after welding without a filler material is a common phenomenon that is attributable to the larger grain sizes in FZ and HAZ [29]. The flow stress increased as the grain size become finer due to grain boundary strengthening [30]. Figure 1 and Figure 7 clearly show the grain growth in HAZ compared with BM. Although a high fraction of LAGB divided FZ into fine regions, Figure 7a shows that the area distinguished by HAGB was much larger than the grain size of BM. Furthermore, LAGB could not act as a strong barrier to the movement of dislocation as HAGB. The elongation to fracture also decreased from 24.9% to 20.2%. This could have been due to the discontinuity of the grain structure between BM, HAZ, and FZ, resulting in residual stresses in the interfaces [31].

When aging treatment proceeded after welding, UTS slightly increased to ~880 MPa, whereas the elongation to fracture decreased to 17.58%. The increase in UTS might be due to precipitation hardening via the Orowan mechanism as a result of carbide precipitation during the aging treatment [32]. Furthermore, Yang et al. reported that precipitates could confine the leading and trailing Shockley partial dislocations depending on the orientation of the stress and its magnitude [27]. Namely, the carbide precipitates could hinder not only perfect dislocation movement but also the slip of partial dislocations. As the deformation mechanism of the Fe-SMA is slip of both the partial and perfect dislocations, both hardening mechanisms could effectively increase UTS.

**Figure 12:**
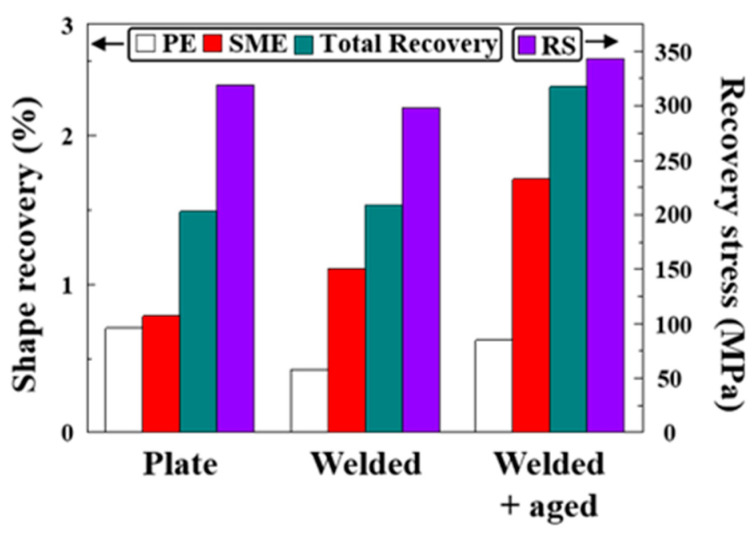
Shape memory performance of the Fe-SMA plate weldments before and after the aging treatment.

Figure 12 shows the shape memory properties of the samples used in this study. After welding the Fe-SMA plates, PE decreased, whereas SME increased. The higher value of PE before welding could be due to the finer grain size of BM than those of FZ, and HAZ. According to Saeed-Akbari et al., Gibbs free energy change for a phase transformation from austenite to ε-martensite in an Fe–Mn–Al–C (wt.%) alloy system is expressed, as follows [33]:

(4) $$\Delta G^{\gamma \to \varepsilon }=X_{Fe}{\Delta G}_{Fe}^{\gamma \to \varepsilon }+X_{Mn}\Delta G_{Mn}^{\gamma \to \varepsilon }+X_{Al}\Delta G_{Al}^{\gamma \to \varepsilon }+X_{C}\Delta G_{C}^{\gamma \to \varepsilon }+X_{Fe}X_{Mn}\Delta \Omega_{FeMn}^{\gamma \to \varepsilon }+\;X_{Fe}X_{Al}\Delta \Omega_{FeAl}^{\gamma \to \varepsilon }+X_{Fe}X_{C}\Delta \Omega_{FeC}^{\gamma \to \varepsilon }+X_{Mn}X_{C}\Delta \Omega_{MnC}^{\gamma \to \varepsilon }+{\Delta G}_{mag}^{\gamma \to \varepsilon }+\Delta G_{ex}^{\gamma \to \varepsilon }$$

(5) $$\Delta G_{ex}^{\gamma \to \varepsilon }=170.06exp\left(\frac{-d}{18.55}\right)$$

Equation (4) implies that the Gibbs free energy change for a phase transformation from austenite to ε-martensite depends on the chemical composition of the given alloy. The last term of Equation (4), ΔG_ex_, represents the excessive energy depending on the grain size (Equation (5)). This equation indicates that the stability of the austenite phase increased as the grain size decreased. Namely, the higher stability of austenite, which resulted from the smaller grain size of the Fe-SMA plate, can promote reverse-phase transformation from ε-martensite to the parent austenite immediately after unloading. Regarding the weldment without the aging treatment, the stress-induced ε-martensite requires heating above the A_s_ temperature to activate reverse-phase transformation. However, the total shape recovery values of the Fe-SMA plate and weldment were similar, indicating that total reversible-phase transformation between austenite and ε-martensite would be similar under given conditions, i.e., 4% prestrain and an activation temperature of 200 °C. Namely, the increase in SME of weldment would be responsible for the change in relative ratio between PE and SME due to the increase in grain size of HAZ and FZ, in other words, the decrease in austenite stability. 

The RS of the weldment before the aging treatment was lower than that of the Fe-SMA plate. Although the RS originated from the force of recovering to its original shape under constrained conditions, the RS value was more dependent on the strength of the material rather than SME during heating [11]. Additionally, Ferretto et al. reported a high RS value for dual-phase Fe-SMA fabricated by L-PBF [34]. They embodied a dual-phase structure comprising ferrite and austenite using site-specific laser rescanning. Although the dual-phase Fe-SMA showed relatively poor shape recovery behavior because the ferrite did not participate in shape recovery, it exhibited high RS. Therefore, the post-welding decrease in RS might have been due to the decreased strength of the weldment than the plate despite the higher SME.

Notably, PE, SME, and RS increased after the aging treatment, and the increases in PE and SME were ascribed to carbide precipitation. Kajiwara et al. reported that carbide precipitation could form a large number density of stacking faults owing to the lattice mismatch between carbide and the matrix [4]. According to them, the stacking faults can act as an easy nucleation site for austenite–ε-martensite phase transformation. Therefore, carbide precipitation enhances SME. Other extant studies also confirmed the beneficial effects of precipitation on SME using various types of carbide and nitride precipitations [16,35,36,37]. The increase in RS was also attributed to precipitation hardening. As already discussed, RS highly depends on strength. Therefore, the enhanced post-aging treatment strength could result in increased RS. The strength and RS somewhat dropped after welding, and the findings regarding aging treatment indicate that the decreases can be overcome by aging treatment for the VC containing Fe-SMAs. 

**Figure 13:**
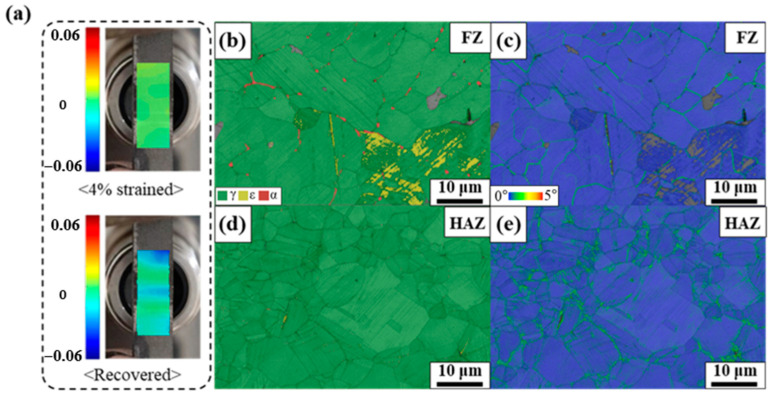
(**a**) DIC analysis results of the weldment without aging treatment before and after activation at 200 °C. (**b**–**e**) phase and KAM map after activation at 200 °C. (**b**,**c**) FZ. (**d**,**e**) HAZ.

Figure 13a shows the DIC analysis results before and after activation at 200 °C. The picture on the upper side shows the local strain after a 4% prestrain. The prestrained state was set as the initial state for DIC analysis, where the local strain was almost zero. The picture on the bottom shows the local strain after heating and cooling, i.e., SME activation at 200 °C. The map of the local strain indicates the decreased variations in the local strain values on both sides of the central area, indicating decreased SME. The area with low SME could correspond to HAZ, and the central area could correspond to FZ. Considering the higher SME in the weldment (without the aging treatment) than the plate as well as the poor SME in HAZ of the weldment, FZ might have contributed to the increased SME in the weldment. Namely, among FZ, HAZ, and BM, the highest SME might have emanated from FZ. Figure 13b,c also show the fully recovered state in FZ. Although some fractions of ε-martensite remained after the activation (Figure 13b), the KAM map in Figure 13c did not reveal any local misorientation in austenite phase, except for LAGB, indicating a fully recovered state. Figure 13d shows that HAZ also exhibited a fully austenitic structure, with a trace of the phase transformation between austenite and ε-martensite after activation represented by the black straight line. However, the KAM map in Figure 13e shows a local misorientation near GB and the austenite/ε-martensite interfaces, indicating its lower shape recovery behavior than that of FZ.

A possible explanation for the good SME in FZ is its larger grain size than BM. Wang et al. reported that grain refinement reduces the ability to suppress plastic deformation and facilitates stress-induced ε-martensite transformation, resulting in reduced SME [38]. Additionally, the presence of additional GBs from grain refinement increased the constraint effects on martensitic transformation. In this study, FZ exhibited a larger grain structure than BM, although each grain was subdivided by LAGBs. As the LAGB can act as just a soft barrier to the movement of partial dislocation and even the dislocation source (might be partial dislocation in this case of such alloys with low stacking fault energy) [23], subdividing each grain by LAGB would not hinder the phase-transformation behavior between austenite and ε-martensite.

**Figure 14:**
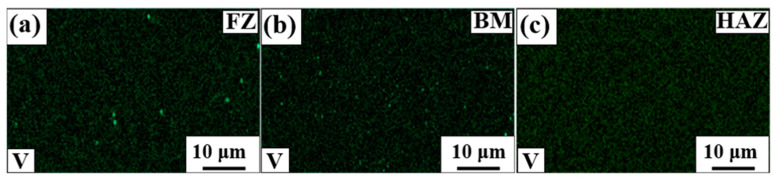
Vanadium element mapping results of the weldment before the aging treatment. (**a**) FZ, (**b**) BM, and (**c**) HAZ.

However, the effect of the grain size on SME cannot fully clarify the poor SME in HAZ, as HAZ also exhibits a much larger grain size than BM. The alloy employed in this study contains V and C, which can form carbide during aging treatment. As aforementioned, carbide precipitation exerts beneficial effects on SME and RS. Figure 14 shows the V-element mapping results of the weldment before the aging treatment. Figure 14a,b show significant evidence of the V-rich precipitates, and the precipitates correspond to VC. The V-rich precipitates were presumably formed during sample preparation and welding. Conversely, there was no evidence of precipitates in HAZ. This could be ascribed to the fact that the pre-existed precipitates dissolved in the matrix when the HAZ was affected by heat during welding. The limited amount of the ε-martensite in HAZ after aging treatment (Figure 9f) also indicated that the precipitation occurred less in HAZ than in FZ and BM, as more energy would be required for the nucleation and growth of the precipitates in HAZ. Kajiwara et al. reported that the NbC precipitation increased SME by >70% compared with the solution-treated state [4]. Figure 12 also shows that the aging treatment increased SME by ~54%, indicating that the presence of precipitates significantly affected SME. Therefore, the poor SME in HAZ could be ascribed to the absence of precipitates, although the grain size of HAZ is much larger than BM (Figure 4). The more precipitates in FZ than in HAZ can also be responsible for the higher hardness value of FZ than HAZ (Figure 6).

Dissimilar to the extant studies, SME in this study increased after welding [16]. The main difference between previous studies and this one is the prewelding aging treatment. In those studies, the aging treatment was performed under the hot-rolled condition before welding to improve SME, i.e., SME was maximized before welding. In that case, the dissolution of the precipitates in HAZ during welding could decrease SME (Figure 13a). The precipitates in FZ could also be dissolved in the matrix because the FZ was completely molten. Although some amounts of the precipitates could be formed during cooling (Figure 14a), SME would not be as strong as that obtained in the aged state (Figure 12). Furthermore, although the previous studies did not state the average grain size of BM, it was not significantly smaller than that of FZ. Stated differently, there was no noticeable grain coarsening in FZ, indicating that the dissolution of the precipitates was the most crucial factor affecting SME. Conversely, the alloy was not aged before welding in this study, although some amounts of the precipitates were already formed. Furthermore, the grain size of BM was much smaller than that of FZ, indicating that the grain coarsening in FZ could improve SME collaborating with the formation of precipitates during welding and subsequent cooling. This finding implies that the shape memory behavior of weldments can be different whether carbide forming elements are contained or not. Furthermore, as the precipitation of carbide during welding process has a crucial role in shape memory performance, the relationship between heat input and precipitation behavior should be further investigated to optimize Fe-SMA welding. 

## 4. Conclusions

In this study, the shape memory and mechanical properties of welded Fe-SMA plate using GTAW were investigated. The optimal welding condition was determined by varying the welding speed and current. Subsequently, the microstructure of the weldment with the optimal welding condition was investigated to determine the relationship between the microstructure and physical properties. The effects of post-aging treatment on the microstructure and properties were also investigated. Additionally, the contributions of FZ, HAZ, and BM to SME were investigated by DIC analysis, and the highlights of this study are summarized as follows:

1. A heat input below 0.12 kJ did not facilitate the full penetration of the Fe-SMA plate with a thickness of 2 mm. All three samples with a heat input of 0.12 kJ but different welding speeds and currents exhibited similar microstructures and Hv values.
2. The solidification sequence under the optimal welding condition was in the order of columnar dendritic ferrite and equiaxed dendritic austenite. However, a fully austenitic structure emerged after cooling. The equiaxed structure in the center region of FZ contained a high fraction of LAGB. The ε-martensite phases were formed by carbide precipitation during aging treatment, although no noticeable changes were observed in the grain morphology.
3. The UTS and elongation to fracture decreased after welding owing to grain coarsening and stress accumulation. Conversely, the aging treatment caused an increase in the UTS, scarifying some amounts of elongation to fracture due to precipitation hardening.
4. PE and SME showed opposite post-welding trends. The PE values decreased owing to grain coarsening in FZ and HAZ, thus decreasing austenite stability, although SME increased by 40%, indicating that heating was essential for the reverse-phase transformation from ε-martensite to austenite. However, the total shape recovery was similar, indicating that the total reversible-phase transformation was similar. The decrease in RS was attributed to the decreased strength of the weldment. On the other hand, aging treatment noticeably improved all PE, SME, and RS via the precipitation behavior.
5. The DIC analysis revealed that FZ contributed the most in improving the SME, and this could be attributed to grain coarsening. However, the contribution of HAZ to SME was even less than that of BM, probably owing to the absence of precipitates in HAZ despite the larger grain size in HAZ than BM.

Because the optimal condition found in this study is the minimum condition to ensure a fully bonded state, that condition does not ensure the best performance of welded-Fe-SMA. Therefore, further study for finding the optimal condition is needed to improve shape memory and mechanical properties. 

## Figures and Tables

**Table 1:** Welding conditions.

Welding Speed (cm/min)	Current (A)	Heat Input (kJ/mm)	Sample Notation
30	53/26.5	0.08	**08-30**
	67/33.5	0.1	**10-30**
	80/40	0.12	**12-30**
40	72/36	0.08	**08-40**
	90/45	0.1	**10-40**
	106/53	0.12	**12-40**
50	90/45	0.08	**08-50**
	112/56	0.1	**10-50**
	134/67	0.12	**12-50**

## Data Availability

The original contributions presented in the study are included in the article, further inquiries can be directed to the corresponding authors.
